# Connected, digitalized wire arc additive manufacturing: utilizing data in the internet of production to enable industrie 4.0

**DOI:** 10.1038/s41598-025-15250-y

**Published:** 2025-11-05

**Authors:** Samuel Mann, Jan Pennekamp, Muzaffer Ay, Mohamed Behery, Lukas Oster, Benjamin Ebert, Rahul Sharma, Dirk Abel, Gerhard Lakemeyer, Uwe Reisgen, Klaus Wehrle

**Affiliations:** 1 Welding and Joining Institute, RWTH Aachen University, Aachen, Germany; 2 Communication and Distributed Systems, RWTH Aachen University, Aachen, Germany; 3 Institute of Automatic Control, RWTH Aachen University, Aachen, Germany; 4 Knowledge-Based Systems Group, RWTH Aachen University, Aachen, Germany

**Keywords:** Mechanical engineering, Electrical and electronic engineering, Computer science

## Abstract

This work explores the potential of connected, digitalized Wire Arc Additive Manufacturing (WAAM) within the framework of Industrie 4.0, analyzing it through distinct process layers: workpiece, assembly, and product. Each layer presents unique timeframes and stakeholder interactions, necessitating varied data infrastructure demands, including a consideration of data security and privacy challenges. The workpiece layer mostly covers the local production setup and is thus directly coupled with the product and process quality as well as maintaining a safe operation. In the assembly layer, ensuring interoperability among diverse stakeholders is crucial, requiring clear definitions of responsibilities and access rights to enhance data exchange. The product layer prioritizes the reliability and trustworthiness of information for informed decision-making, advocating for solutions that guarantee authenticity and verifiability while addressing privacy concerns through techniques like privacy-preserving computing. The paper identifies a critical gap in real-world applications of these concepts in additive manufacturing. It proposes a data-driven quality control approach to enhance process and product quality in arc welding, leveraging digital shadows to create effective interfaces within production networks. This approach has demonstrated potential reductions in welding fume emissions by 12–40%, alongside connected applications that minimize exposure and energy consumption.

## Introduction

Connected, digitalized manufacturing has been the focus of well-known initiatives such as Industrie 4.0, etc., for several years now and allows a variety of new perspectives on process optimization through supply chains and product life cycles^1–6^. Corresponding concepts of production technology describe manifold potentials but often also raise a multitude of further questions.

Additive manufacturing and specifically WAAM come with strong fundamentals due to already digitalized process chains. According to ISO/ASTM 52900:2021^7^, WAAM is classified as a Directed Energy Deposition (DED) process, which the standard defines as an “additive manufacturing process in which focused thermal energy is used to fuse materials by melting as they are being deposited”. In this case, the electric arc serves as the focused thermal energy source.

However, WAAM faces similar challenges as other production processes. Especially when different subsystems, competencies, and parties join a network of various information providers and stakeholders^8^. At this point, different requirements for time scales in which data must be collected and passed on collide. Given the sensitivity of the collected data, paired with the interconnectedness of assembly lines and production sites, data security and privacy aspects have to be considered as well^9^.

As core components of digitalized production systems, cyber-physical systems (CPS)^10^, vertical and horizontal networking^2^, data analytics^1^, and digital engineering^11^, are usually mentioned. Digital representations of production processes can either be a digital twin^12^ or a digital shadow^13,14^ of the process. Digital shadows represent a more focused alternative to digital twins, capturing only the most relevant aspects of a physical system for a specific task, such as quality prediction, while maintaining computational efficiency for real-time applications. The former is the process’ digital counterpart and can be used to predict next production steps or machine calibration for a new product (e.g., FEM simulations). The latter is a compact model that represents only a specific part of the physical model aimed at a specific task (e.g., production quality prediction)^15^.

Nevertheless, these terms still represent abstract concepts in applications for which few comprehensive implementations exist. Work on CPS in the context of WAAM or arc welding is not uncommon, but it also describes a variety of different basic understandings and applications that make further collaborative development and implementation difficult.

In this paper, we apply the process layer framework^16^ to WAAM, organizing our analysis along three distinct layers: workpiece, assembly, and product. Each layer represents different timeframes of operation, involves distinct stakeholders, and faces unique security and privacy challenges. By examining WAAM through this structured approach, we can better address the requirements for comprehensive data acquisition and quality control. Following this analysis, we demonstrate how data-driven quality control creates a practical implementation path for these concepts, enabling enhanced process quality while addressing security concerns.

## Results

Our research presentation consists of two main parts. First, we will introduce the different process layers that are involved in WAAM to give a detailed overview, i.e., workpiece, assembly, and product process layer. Specifically, we discuss the different timeframes and involved parties to highlight the characteristics of WAAM. Second, we focus on data-driven quality control in this context to improve the state of the art in WAAM by utilizing networked production technology.

### Process layers

**Figure 1:**
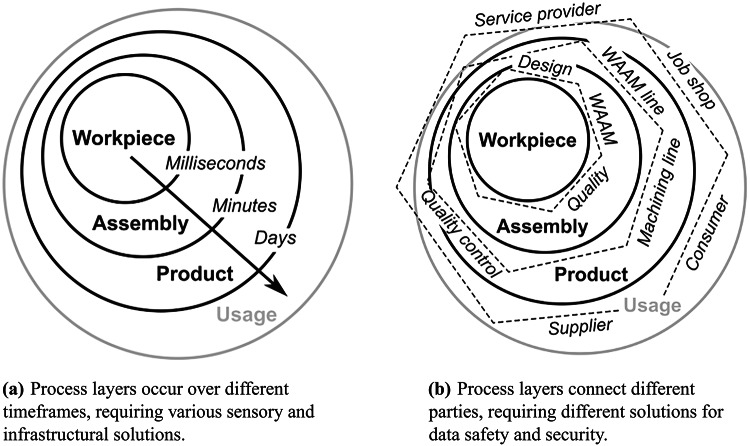
Process layers cover different timeframes and involve several parties.

Following earlier work^16^, WAAM can be understood as organized in concentric process layers, as illustrated in Fig. 1. At the core is the workpiece process layer, where the arc process melts the wire electrode into a geometrically defined shape. This layer directly involves design, production, and quality monitoring, with events occurring in milliseconds. The assembly process layer encompasses the workpiece within a broader manufacturing context, including multiple processes like mechanical processing and coating across several departments. Finally, the product process layer situates the assembly within the overall supply chain, connecting various suppliers, service providers, and customers.The timeframes expand from milliseconds at the workpiece layer to hours or days at the product layer, while the number of stakeholders increases at each level.

These process layers differ not only in timeframes but also in the parties involved and their security requirements, as shown in Fig. 1a, b. This layered perspective allows us to examine the specific challenges and solutions for each context within WAAM implementations.

The application of concepts under the term Industrie 4.0 or Internet of Production (IoP) propagates ubiquitous data transfer and connection but usually does not distinguish between each layer^17^. Nevertheless, each process layer has its own specific challenges and peculiarities. Figure 1 separates these peculiarities in timeframes and involved parties.

The relevant timeperiods vary from milliseconds for the AM workpiece to days in the context of entire supply chains. Data acquisition and corrective intervention require fundamentally different technical approaches. Furthermore, especially with the external process layers, the ability to communicate between different parties becomes increasingly important.

Data-enhanced quality control approaches can bridge this gap in timeperiods by said data and therefore transcend the different process layers. The quality control has the duty to manipulate the core process of wire electrode remelting such that the surrounding quality criteria are met, although the quality can only be determined at a much later time. However, for most processes, surrogate criteria and models can be found to determine the relationship between core process characteristics and the resulting quality of the final product with the help of data. This way, the geometric quality during milling can be manipulated by controlling the active force or the cut face roughness during laser cutting according to the cut kerf width^18^. These surrogate models can also be described as digital shadows since they are describing the data from a defined perspective with adjusted functional proportionality between accuracy and complexity. Moreover, in the specific case of WAAM, the geometric quality and fume emissions can be linked to the power supply of the process by appropriate models / digital shadows and thus be controlled by model-based control approaches. Model predictive control (MPC) specifically combines model-based and optimal control at the same time^19^. MPC, therefore, allows companies to maximize productivity, while the quality constraints and criteria are met along the way during the process^20^.

Finally, aspects related to (data) security and privacy further have implications on these layers. While these aspects are primarily concerned with safeguarding sensitive data that captures intellectual property and may provide insights into respective operations, an appropriately secured production site is essential to ensure a safe operation. The following subsections discuss means and best practices when securing the corresponding process layers for a reliable data-driven WAAM.

#### Workpiece process layer

**Figure 2:**
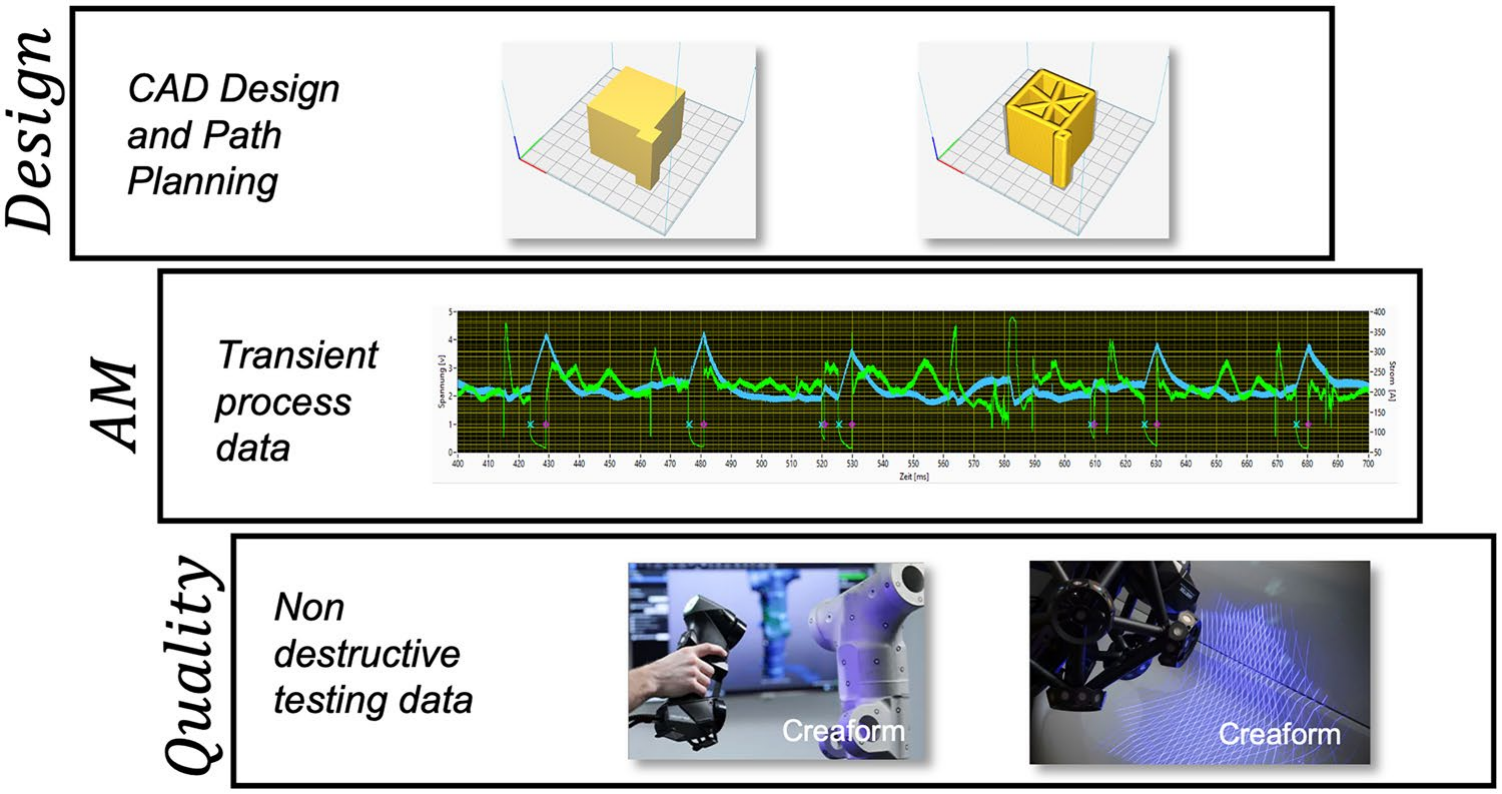
The weld seam process consists of three different substeps: (i) the requirements are set as part of the design, (ii) the AM process is monitored as part of the AM, and (iii) the result is tested as part of the Quality component.

The first and most prominent process layer can be described as the workpiece process layer, which is illustrated in detail more detail in Fig. 2. Digital AM process chains provide a CAD model or at least some kind of path planning that contains valuable information about the target geometry.The AM process, on its own, provides process data that can be used to gain quantified information about the current product and process quality.Finally, means of digital, non-destructive quality testing such as 3D scanning provide the fundamentals to acquire data sets for supervised learning.These opportunities are especially accessible for WAAM compared to fusion welding, which may not be digitalized entirely yet. Events that have a decisive impact on product quality oftentimes occur in the duration of milliseconds and, therefore, require fast data acquisition, e.g., of process current and voltage and the capability to process large amounts of data.

Current situation On the workpiece process layer, a primary security aspect also concerns the safety of the process^21^. Following the interconnected nature of modern manufacturing sites, as required by interconnected production devices, cells, and sites in an IoP^22,23^, sufficient security measures must be in place to allow for a smooth operation. In particular, the safety of the production environment is a crucial aspect, i.e., protecting the workforce, the environment, and the production device from any harm. Recent cyberattacks have shown the great threat of insufficiently secured industrial production devices^24^. Studies further show that even though secure protocol variants are available, they are rarely used and, at times, even configured insecurely^25–27^. Apart from the aforementioned threat concerning safety, insecurely secured devices also open the room for information leakage, negatively impacting the involved companies’ privacy and operation. On the workpiece process layer, this issue particularly concerns the producing company as well as the device manufacturer^8^. While the former risks the unintended distribution of sensitive product designs and production steps, which is a crucial concern in the context of AM—based on known designs, products can be replicated quite easily—the latter can be impacted by unauthorized access to usage information, tool wear, and others. To summarize, while the ability to exchange information and process data in real time promises to improve production processes greatly and product quality, security and privacy aspects require careful consideration.

Security recommendation To address this situation and to enhance the robustness of WAAM production lines, several best practices should be followed. These recommendations can, for example, follow recognized standards, such as IEC 62443^28^ for industrial automation and control systems, NIST SP 800 series^29^ for cybersecurity controls, and ISO/IEC 27001^30,31^ for information security management. In particular, the following recommendations are crucial for the workpiece layer.

- *Harden device configurations and communication:* Devices on the shopfloor, which also covers the workpiece layer, must enforce client authentication and implement fine-grained access control. These configurations prevent unauthorized (third) parties from accessing sensitive information. Moreover, without access to a specific device, they cannot communicate with other devices through said device on the assembly layer. Role-based (RBAC)^32^ or attribute-based (ABAC)^33^ access models are examples that are worthwhile to be integrated. Overall, this measure improves the security configuration over insecure (legacy) deployments.
- *Prohibit the reuse of security secrets:* Security secrets, such as credentials or cryptographic material, may not be reused across devices, cells, or production sites since comprising a single secret then has extensive implications. Most importantly, externally-sourced security secrets should never be reused to prevent reliance on untrusted secrets that may already be compromised^26^. Moreover, deployed security secrets must be treated with caution, i.e., this sensitive information may never be accidentally disclosed (e.g., via container registries or code repositories)^34^.
- *Secure legacy devices:* Retrofitting legacy devices with appropriate security mechanisms is crucial for maintaining a safe and secure operation^35^. Novel architectures promise to equip existing communication infrastructures with the required security mechanisms, even retroactively^36^. In addition to maintaining compatibility, such evolutions are ideally transparent in providing required security mechanisms, avoiding a cost-sensitive replacement of legacy devices.
- *Enable continuous monitoring and logging:* To support proactive threat-hunting activities as well as retroactive incident reports, the workpiece layer should keep detailed logs (in real time).
- *Training operators:* Operators should repeatedly receive training on security matters to behave according to the aforementioned recommendations. This way, they may even identify insecure practices which can support threat hunting.

Moving on, we next focus on the assembly process layer environment.

#### Assembly process layer

**Figure 3:**
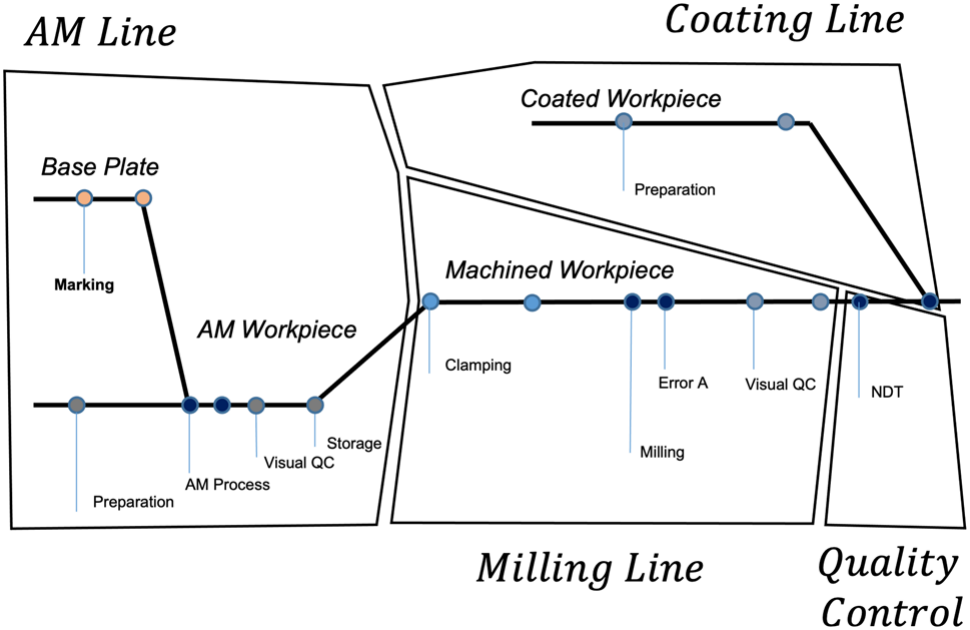
The assembly process maps the workpiece flow to all departments (e.g., AM-, machining-, coating-line, and quality control) that are involved during production.

The second introduced layer consists of the assembly process. Figure 3 describes the workpiece flow of a corresponding assembly process over different departments and individual steps until the assembly is finalized. All steps are defined according to operational departments and responsibilities regarding their competence limits. The flow of information follows the workpieces to the assembly and is described by events. In addition to the quality optimization of the workpiece layer described before, process transparency and optimization are becoming increasingly important at this level. However, the challenges not only result from recording the corresponding events and corresponding workpieces but also in the interfaces between the various competencies limits.

Current situation With the move toward smart manufacturing and digital factories, the privacy issues on the assembly process layer have increased significantly as well. While previous production sites were only operated by a single stakeholder, the transition to shared resources also calls for isolation of associated process data, especially when multiple stakeholders are involved in the assembly process. From process and business points of view, these changes are desirable as they promise to reduce unnecessary redundancy while improving the output quality due to the stakeholders’ focus on specific tasks and their core expertise. However, privacy-wise, involved companies need to carefully gauge the level of shared information on their assembled product, their production history and data, and process details to reduce the risk of exposing sensitive details. Likewise, deployed solutions need to ensure interoperability of data and communication standards to open the assembly process layer to different stakeholders^37^. Accordingly, novel security architectures are needed to support the transition toward smart manufacturing as today’s approaches are primarily concerned with external attackers and the security of a single, specific stakeholder. Unfortunately, these assumptions do not hold anymore in modern production environments.

Security recommendation Challenges on the assembly process layer complement the concerns outlined before, such as the need for secure device configurations, reliable secret management, and secured legacy devices. However, the communication within this layer introduces additional challenges that could be tackled through the following practices.

- *Deploying secure-by-design protocols:* In light of increasing data sharing among stakeholders, communication between assembly devices should follow the secure-by-design^38^ principle. Corresponding protocols, such as OPC UA^39^, MQTT with TLS^40^, and Modbus TLS^41^, are readily available and offer encrypted, authenticated channels^25,26^ that protect sensitive data of the assembly process layer as it flows across the different devices. Their adoption is essential when creating a secure assembly process layer.
- *Introducing security gateways:* Just like with retrofitting devices, securing parts of the assembly process layer with security gateways is another option. This approach does not directly impact existing deployment but may instead reliably enforce encryption and authentication, similar to the retrofitting measures discussed for the workpiece process layer. This approach ensures compatibility and security even when existing hardware cannot be immediately replaced.
- *Collaborative security configuration:* If involved in a multi-stakeholder deployment, maintaining a secure security configuration is a joint effort, i.e., the setups should be aligned and interoperable. Optionally, stakeholders may even join their logs to ease the analysis of security-relevant events (across their deployments).

In summary, while many foundational security principles, such as secure device configuration and communication, hold on both the workpiece and assembly process layers, the (supported) multi-stakeholder nature of the assembly process layer introduces additional challenges related to data security and privacy.

In the next subsection, we continue our presentation of the different process layers with the product process layer.

#### Product process layer

**Figure 4:**
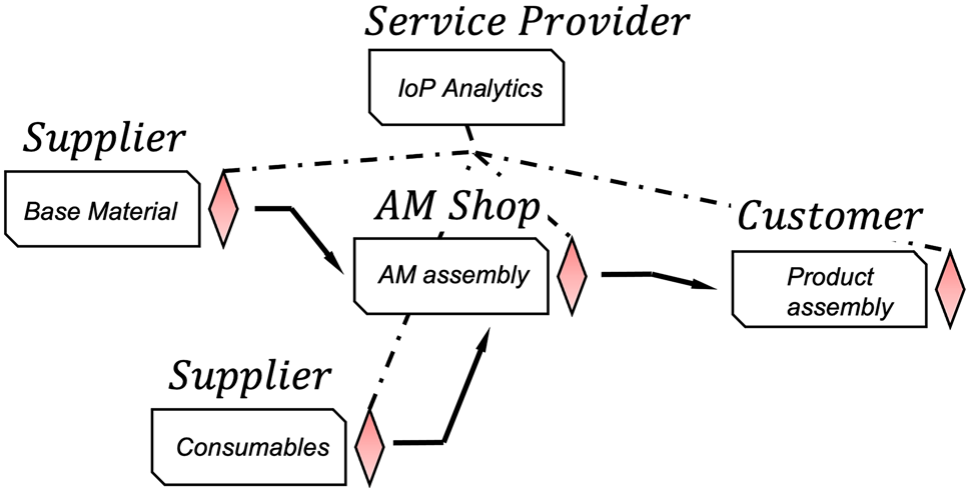
The product process entails different parties (suppliers, welding shops, service providers, and customers) and their products until the final good is produced.

The third process layer describes the overall product process. Figure 4 on the right shows the various parties in the supply chain interaction to realize the final product based on the AM assembly. In this process layer, for example, material properties of current batches of the filler material can be utilized during AM process optimization and quality control. Another benefit is the communication of the realized actual geometry with the customer, who might carry out additional post-processing or assembly. In addition, IoP service providers in the supply chain could potentially offer data analytics. However, they would need access to production data. The need for data security and privacy becomes especially clear at the latest with this process layer because information must be communicated beyond company borders. In contrast, corresponding product processes take longer, which offers additional opportunities for secure communication solutions.

Current situation The product process layer is highly relevant and interesting from a security and privacy perspective. As for the previous two layers, the interconnection of various companies that are not sourcing the same production site introduces new challenges. First, previously isolated production sites are now connected to the Internet, allowing for improved production processes by sharing relevant production data. Moreover, details on the operation and scheduling of production processes and deliveries allow companies to improve the corresponding management, also on site^42^. In this setting, data sharing is mostly related to existing supply chains. However, it can involve both horizontal and vertical collaborations. In this context, the reliability and trustworthiness of information are particularly important to allow for well-founded decision-making. To this end, companies are interested in technical approaches that guarantee the authenticity and verifiability of information, from sensing to storage^43^. Unfortunately, such work is still in its infancy, limiting the straightforward use of external information. Once information has been sensed, companies also need to make sure that it is available long-term and that it can be made available to all relevant parties. Especially when considering dynamic settings like the IoP, not all data-accessing parties might be known upfront. Consequently, corresponding infrastructures must be deployed in the wild to account for these challenges^44^.

Second, when looking for improvements both on a process level but also concerning the product’s production, companies are interested in external knowledge to avoid costly experiments and missteps. Especially the IoP envisions a global exchange of such data to make knowledge accessible where it is really needed. In part, this idea promises to push new developments to production sites and companies on a global scale. Here, privacy-preserving computing is a key technology that still addresses the privacy needs of participating companies. For example, architectures^45^ or data spaces^46^ for privacy-preserving information exchange are in high demand. Moreover, before the exchange of information, companies need to be aware of the information they are looking for (e.g., through company benchmarking^47^) and how they can access it^8^. In the context of AM, the benefits of information sharing are especially of interest as specific production steps can be realized in many production sites, as AM frequently profits from general-purpose devices that allow for relatively quick production and process changes.

Overall, we identify the need for sophisticated technical concepts that reliably and trustworthily enable the exchange of information between stakeholders on the product process layer. As the development toward an interconnected landscape has just begun, corresponding real-world deployments, especially in the context of AM, are generally missing. Thus, realizing a gradual implementation in a secure manner will be a significant challenge. Finally, when looking beyond the product process layer, we also notice similar challenges arising from the usage layer, i.e., the integration of customers and consumers in the data flows, data sharing efforts, and product, as well as process improvements, is another important future challenge^8^. With an appropriate and secure technical foundation, the connected, digitalized wire arc additive manufacturing will experience great process-related benefits.

Security recommendation As outlined, the product process layer in WAAM introduces a broader and more complex set of security challenges due to its highly interconnected nature. Unlike the more localized exposure in the workpiece and assembly process layers, the product process layer almost always involves the sharing of sensitive information across company boundaries and the integration of external information sources. In line with previously discussed recommendations, the following measures should be considered when safeguarding the product process layer.

- *Follow established security guidelines:* Given the exposure of previously isolated production environments to the Internet, the importance of network security increases. Stakeholders should thus follow configuration best practices as outlined in *NIST SP 800-82*^48,49^ and *IEC 62443-3-3*^50,51^, including segmented networks, intrusion detection, and the deployment of firewalls to restrict unauthorized access.
- *Deploy maintainable infrastructures:* Especially in interconnected environments like the product process layer, deployments should allow for quick updates to enable companies to keep up with the rapid changes in the security landscape. For example, certain cryptographic primitives may become obsolete or insecure over time^52^, endangering long-term confidentiality and authenticity—aspects that may have a significant impact on the operation. Certainly, the previous two recommendations are also paramount for the other process layers.
- *Utilize Virtual Private Networks (VPNs):* Production sites should not be directly exposed to the Internet to reduce the attack surface. Consequently, operators should rely on VPNs to establish connections between production sites over configuring direct access from the Internet. This approach adds an additional layer of security.
- *Setting up privacy-preserving data sharing:* When collaborating with other companies, building blocks that ensure privacy preservation, such as secure multiparty computation (SMPC)^53^ or federated learning (FL)^54^, can help with maintaining control over sensitive information. However, recommendations regarding specific protocols are highly use case-specific and thus are out of scope for this paper.

Having examined the security and privacy considerations across the three process layers, we now turn to the practical implementation of these concepts through data-driven quality control. This approach bridges the theoretical framework of process layers with actionable solutions that address quality concerns while maintaining appropriate security measures at each level.

### Data-driven quality control

Data-driven quality control represents a practical implementation pathway to address the challenges identified across the three process layers. This approach enables cyber-physical systems (CPS) in WAAM by creating interpretable data models that facilitate communication between system components and across organizational boundaries, thereby addressing both quality and security concerns.

According to related work^6^, various core components can be defined for Industrie 4.0 concepts in the context of arc welding processes. Here, CPSs are named as key drivers. According to Bahati and Gill^55^, CPSs are characterized by the “ability to interact with, and expand the capabilities of, the physical world through computation, communication, and control”. Compared to conventional, automated manufacturing systems, the focus here is on the ability to interpret and communicate. Sensor technologies have become very powerful in terms of their basic function. However, sensor data still have to be interpreted with regard to decisive characteristics. In addition, the system components, even in modern additive manufacturing systems, have only limited communication capabilities in the sense of networked production technology.

**Figure 5:**
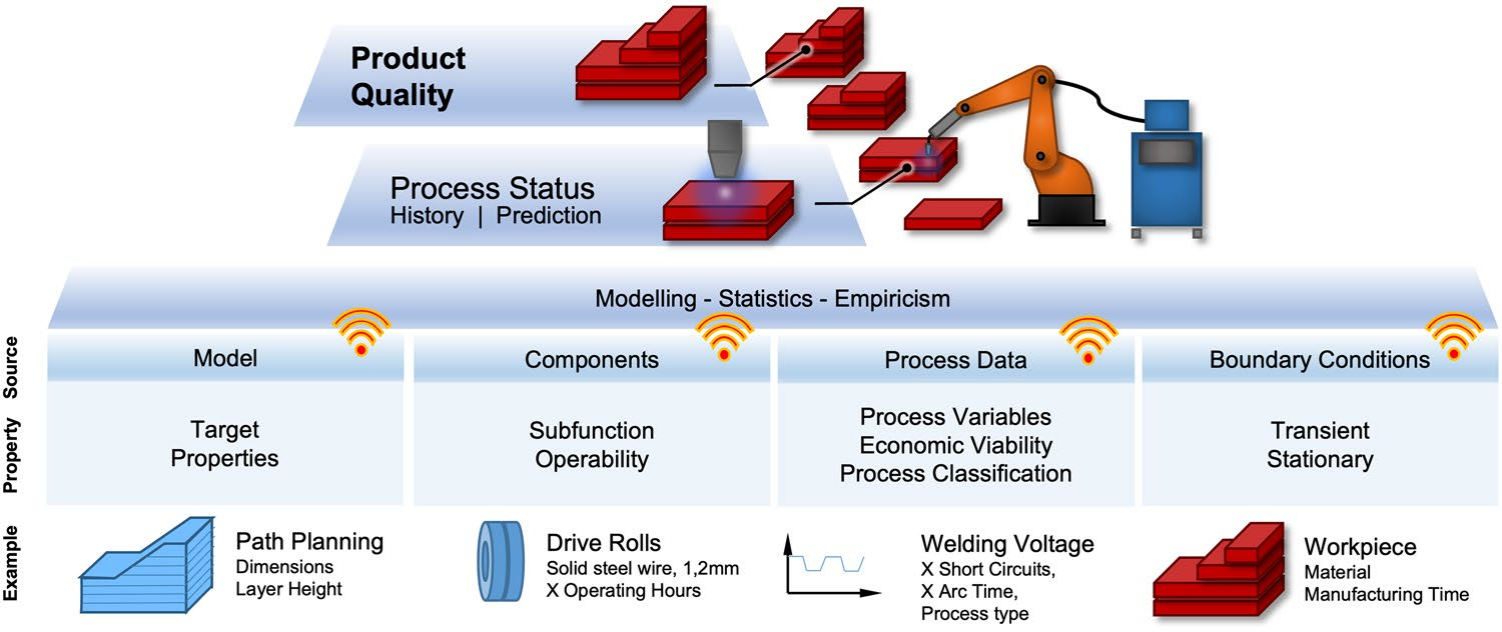
Networked product quality for WAAM according to Jodelbauer^1^.

**Figure 6:**
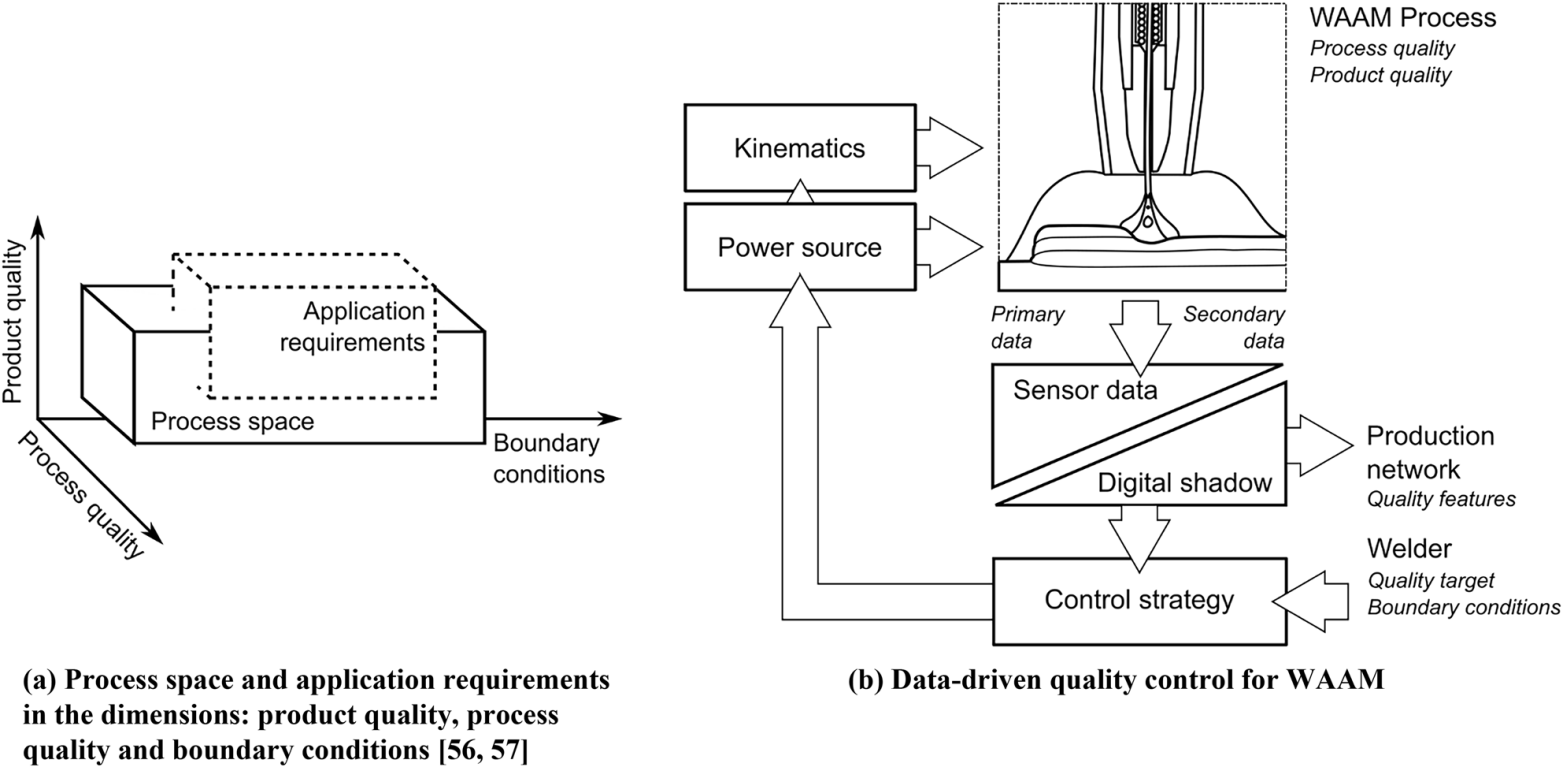
Fundamental concepts of data-driven quality control for fusion and additive arc welding processes according to prior work^57^.

Figure 5 motivates, based on previous work^6^, different information sources and their exemplary properties of WAAM, which can serve as a valuable basis for further data-based interpretation possibilities. Thus, on the one hand, a statement about the history of the process state and the potential for the prediction can be uncovered. The target in Fig. 5 is, however, a statement about the result of the WAAM process—the product quality. To summarize basic target features in the context of arc-based manufacturing processes, two quality dimensions can be defined first, according to prior work^56^ in Fig. 6a. These features include the process quality with the volatile process properties, e.g., economic and sustainable characteristics such as process emission. The product quality, on the other hand, describes the non-volatile properties of the workpiece, e.g., geometry and mechanical properties. However, both quality dimensions must be considered in conjunction with the boundary conditions, e.g., filler materials and other welding consumables. The core challenge of welding arc-based manufacturing is thus to match the application requirements space with the available process space.

This abstract but fundamental challenge is currently solved by human process competence, which has become increasingly rare. However, connected production environments and concepts like the IoP or Industrie 4.0 create the potential to shift this competence into the manufacturing system, to optimize production, and to support the personnel.

Recent studies^57–67^ demonstrate existing approaches for quality control in gas metal arc welding (GMAW), particularly using imaging sensors.

Moreover, ongoing WAAM monitoring advances have improved geometric accuracy and material properties. Scheck et al.^68^ developed closed-loop control for weld pool size, compensating for increasing interpass temperatures and improving geometric accuracy. On a different note, Treutler et al.^69^ demonstrated how melt pool size control affects cooling conditions and microstructure in low-alloy steels, enhancing mechanical properties.

However, clear challenges remain: Several works^58–60^ describe process-oriented modeling for quality control and novel sensors but closed-loop control system remain rare, especially using data-driven modeling approaches.

In fact, the process stability and repeatability in WAAM are highly dependent on temperature control during manufacturing. Various approaches for temperature monitoring in WAAM have been investigated in recent literature. For example, Jorge et al.^70^ distinguish between two main pyrometric measurement strategies for interlayer temperature (IT) monitoring: the “Upper Pyrometer” strategy, where the pyrometer measures temperature on the top face of the last deposited layer, and the “Sideward Pyrometer” strategy, measuring from the lateral side of the wall. Müller and Hensel^71^ demonstrated that emissivity during WAAM of high-strength steel depends on both temperature and surface purity, making calibration essential for reliable measurements. For aluminum alloys, Vazquez et al.^72^ applied thermography to optimize interpass dwell times by establishing maximum local temperature thresholds to avoid segregation-related problems.

Notably, past research^61–63^ successfully demonstrated closed-loop control systems but for less demanding tungsten inert gas (TIG) welding processes. GMAW requires more robust optical sensors due to dynamic lighting conditions, fume emissions, and spatter formation. In prior work^57^, a general approach for data-driven quality control was introduced for GMA welding, but it lacks application for wire arc additive manufacturing.

Figure 6b shows the concept of data-driven quality control, which offers a technical concept to solve Fig. 6a. Based on highly available primary sensor data (e.g., process current and voltage) and secondary, significant sensor data (e.g., process images), the model-based digital shadow can make a quantifying statement about the product or process quality. At this point, the manufacturing system gets an essential communication interface with the production network. Compared to the digital twin, the digital shadow is a sufficiently precise representation that allows short-term calculation in the context of closed control loops. The subsequent control strategy moderates any conflicting goals, especially with basic process stability, and is able to influence the additive manufacturing process via the power source and kinematics. At this point, this concept not only delivers quantifying values about the process and product quality but also closes the quality control loop.

The concept of data-driven quality control thus provides the manufacturing system with all the necessary properties of cyber-physical systems. This concept works with process data on the workpiece process layer but also enables meaningful networking in higher-level process layers via the digital shadow and according interfaces. In addition, this interface offers a connection to the World Wide Lab (WWL)^15,21^, which is a central concept of the Internet of Production^21^. It aims at establishing a lab of labs allowing the sharing, storage, and querying of manufacturing data from different production sites. The core idea is to exploit data diversity for higher prediction accuracy^13–15^. Within the WWL, we can extract the digital shadows of the WAAM process (e.g., aimed at quality prediction) and reuse this data not only for process parameterization and control, but also for quality monitoring and inspection. Prototypical implementations of frameworks for sharing and querying the digital shadows were shown in^13,14,73^. They demonstrate the application of the WWL for different industrial use cases. In addition to data storage, they allow different users to analyze the stored data and models as well as provide a decision support system giving feedback to the users for process control.

In studies on the control of the process and product quality, the welding fume emission (process quality) could already be minimized, and the weld seam geometry (product quality) could be successfully controlled by applying data-driven quality control.

**Figure 7:**
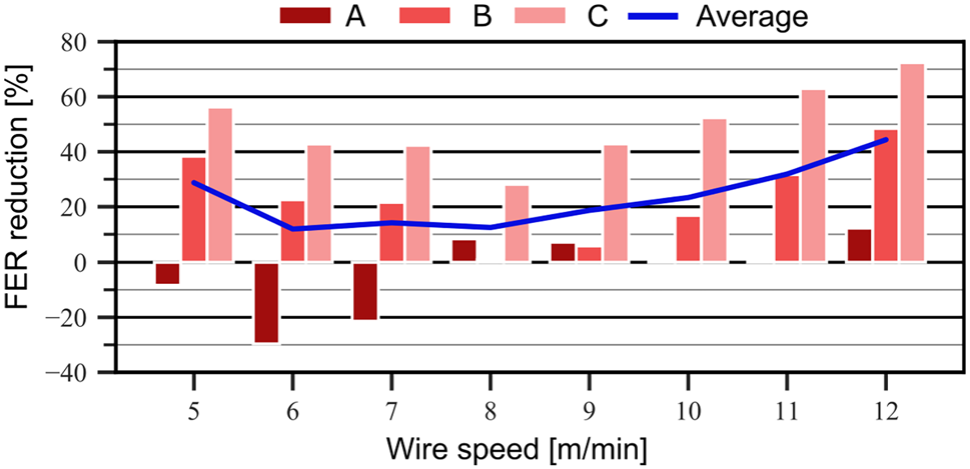
Potentials of fume emission reduction with data-driven quality control^57^.

Figure 7 shows the potential for minimizing the FER when using data-driven quality control. Recently, the fume emission rate (FER) has gained increased attention alongside noise and radiation emissions. In 2018, the International Agency for Research on Cancer (IARC) classified welding fumes as carcinogenic (Group 1). This classification is particularly concerning as recent exposure studies suggest that current protective measures in workplaces are inadequate^74^. Despite existing safety measures, exposure limits for respirable dust (A-dust) and manganese-containing dust often exceed recommended thresholds^75^. This study, however, shows that the FER can be minimized over wide process power ranges of the GMAW process between 12% and over 40%, based on various process parameterizations (A, B, and C). In addition to reducing harmful welding fumes for personnel, the extraction power can also be controlled in a targeted manner. Thus, even for enclosed additive manufacturing cells, the energy consumption of the extraction system can be reduced, which can even exceed the power requirements of the actual welding process.

**Figure 8:**
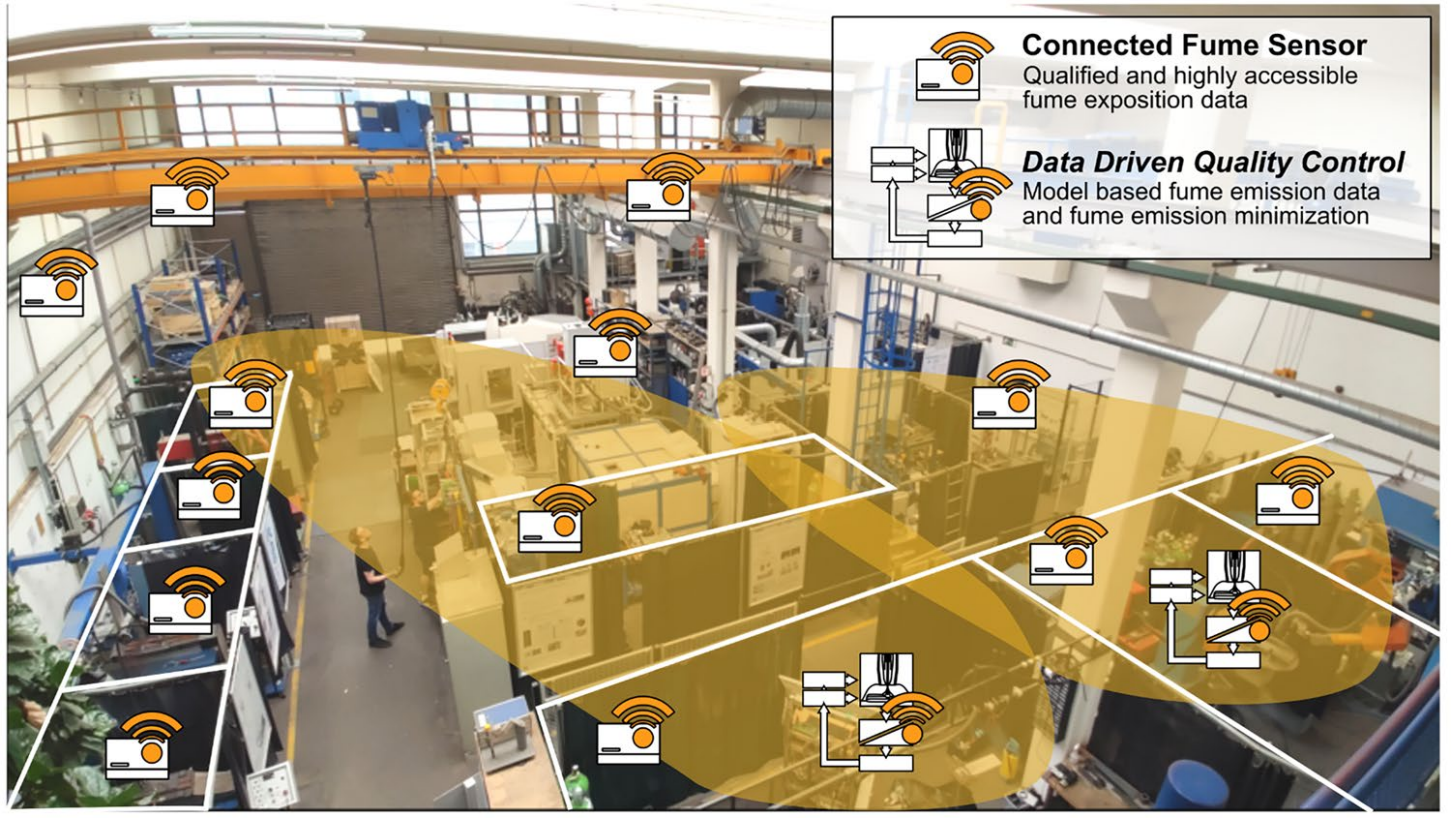
Connected fume sensors and data-driven quality control to model and predict fume exposition in production.

In addition, manufacturing cells can be integrated directly into the same data infrastructure with low-cost, qualified welding fume sensors using the concept of data-based quality control (Fig. 8). Thus, emission models at the point of origin can be coupled with exposure measurements. From this, valuable information about the welding fume exposure in different workplaces, the temporal welding fume distribution in production halls, and potential extraction measures can be investigated and further developed.

## Methods

To implement and validate the concepts discussed in the process layers and data-driven quality control sections, we developed an experimental approach focused on welding fume emission control as a key quality parameter. Our methodology encompasses welding parameters, fume measurement, data acquisition, model development, and control system implementation, providing a practical demonstration of the theoretical framework discussed above.

### Welding parameters

The study to acquire the quality model for fume emission rate (FER) in gas metal arc welding (GMAW) utilized an inverter welding machine (EWM Titan XQ 400) and employed the LCVT algorithm to distribute 240 parameter sets for a standard MIG process. The welding voltage ranged from 15 to 37 V, the welding current from 190A to 410A, and the wire feed speed from 5m/min to 12m/min while maintaining basic process stability. The welding speed was adjusted from 0.4 m/min to 0.68 m/min. The welding wire consists of an EN ISO 14341-A: G 3Si1; $\varnothing =$ 1.2 mm with an ISO 14175 - M21 - ArC – 18 shielding gas, welded on a S235JR (1.0038) base material.

• **Fume emission rate measurement**
  FER measurements were performed for each parameter set using a fume chamber (Fume-box) in accordance with DIN EN ISO 15011-1 standards. This method ensures accurate and standardized quantification of welding fume emissions.

• **Data acquisition**
  Time-series data of welding voltage and current has been recorded at a sampling rate of 100kHz and 16bit resolution. The welding current was measured using a Hall sensor (LEM HTA 500-S) attached to the workpiece cable. For welding voltage measurement, an isolation amplifier (Analog Devices AD215AY) was placed between the workpiece and the welding torch.

• **Model development**
  The welding fume emission model was constructed using multiple linear regression analysis. This statistical approach allows us to correlate FER with features extracted from the welding current and voltage data, providing insights into the relationship between process parameters and fume generation.

• **Quality control system**
  To implement FER control, the arc correction parameter of the welding machine has been manipulated through a robot interface (RintX12). This interface allowed the adjustment of the welding parameters in real time using an analog output signal. The control loop was completed with a proportional-integral (PI) controller, enabling dynamic regulation of the fume emission rate during the welding process.
  We refer to prior work^57^ for additional details on related methods in the area.

## Discussion

Our research on connected, digitalized Wire Arc Additive Manufacturing (WAAM) contributes to the evolving landscape of Industrie 4.0 by approaching the manufacturing process through distinct process layers. This approach aligns with and extends previous work on layered manufacturing systems. While existing literature often treats digitalized manufacturing as a monolithic concept^1–6^, our process layer framework provides a more nuanced understanding of the different requirements, timeframes, and stakeholders involved at each level.

Compared to the current state of the art in WAAM research, which has primarily focused on technical aspects like process parameters, material properties, and geometric accuracy^69,70,72^, our work expands the discussion to include the critical data infrastructure requirements across different process layers. Recent reviews for arc welding technologies^6,16,56,57^ have identified digitalization as a key future direction, but concrete implementations remain scarce for WAAM, particularly regarding security considerations and quality control in networked environments.

The workpiece process layer presents unique challenges related to millisecond-level data acquisition and processing, which aligns with findings from recent studies on high-frequency monitoring in welding processes^59,60^. Our approach extends this work by addressing the security implications of such high-frequency data acquisition in modern production environments, an aspect largely overlooked in existing WAAM literature.

### General security recommendations

Security and privacy considerations are essential in securing and safeguarding WAAM operations across all three process layers. Given the rising interconnectivity, multi-stakeholder collaboration, and real-time data flows, the complexity of this aspect increases significantly. While each layer introduces unique risks, ranging from local device manipulation over operational safety concerns to information leakage, they all demand a unified and future-proof security strategy. As a key takeaway, we want to highlight two aspects: correct security configuration and continuous monitoring of the situation.

First, deployments must be configured according to protocol-specific security guidelines, such as those best practices defined for TLS^76^, OPC UA^39^, and MQTT^40^. Secure communication depends on the appropriate choice of cryptographic primitives and key material to ensure a secure and reliable operation of encryption, authentication, and access control mechanisms. These technical safeguards must be complemented by adherence to general best practices from respected entities like NIST^77^ and BSI^78^. Misconfigurations continue to be a leading cause of vulnerabilities and, in turn, security incidents.

Second, maintaining a secure operation constitutes an ongoing process. Even well-configured systems can degrade in effectiveness over time due to evolving threats, compromised cryptographic primitives, or other identified weaknesses. Especially in WAAM, where hardware often remains in use for decades, continuous monitoring, risk reassessment, and the ability to adapt to changing conditions are critical activities for every deployment. Without this approach, specific devices or even entire production sites may unknowingly become entry points for attackers. Thus, this aspect is directly linked to the previously raised issue of misconfigurations.

Ultimately, a secure WAAM environment should capitalize on standards, such as IEC 62443^50^, ISO/IEC 27001^30^, and NIST SP 800-82^49^, to implement a secure environment. Nonetheless, real-world deployments require an ongoing and careful assessment to ensure the desired security guarantees.

### Identified synergies

In the context of data-driven quality control, our approach advances the current state of the art by implementing closed-loop control systems for WAAM processes. While recent work has explored various sensing technologies^58–60,71^, effective closed-loop control approaches, particularly for gas metal arc welding processes, are still rare^69^. Closed-loop control mechanisms have shown promising results in improving geometric accuracy and process stability across various additive manufacturing techniques^68^. The reduction in welding fume emissions achieved in our implementation (12–40%) represents a significant improvement over existing techniques, addressing a critical health and safety concern highlighted in recent occupational health studies^74,75^.

The digital shadow concept that we have implemented aligns with emerging trends in digital twin technologies^12–14^, but our approach is distinguished by its focus on practical implementation in WAAM production environments. Unlike theoretical digital twin frameworks often discussed in the literature, our digital shadow represents a lightweight, task-specific model with demonstrated real-world benefits in terms of process quality and emissions reduction. Our work connects to the broader Industrie 4.0 and Internet of Production concepts by creating effective interfaces for data exchange across process layers, addressing a gap identified in recent reviews of cyber-physical systems in manufacturing^24,55^. The lack of comprehensive implementations of these concepts, particularly in additive manufacturing contexts, underscores the novelty and significance of our approach.

The transition from the discussion of process layers to their practical implementation through data-driven quality control demonstrates how theoretical frameworks can be operationalized to address real-world manufacturing challenges. This endeavor bridges the gap between abstract Industrie 4.0 concepts and practical applications in WAAM production environments.

## Conclusion

Our research on connected, digitalized Wire Arc Additive Manufacturing (WAAM) within the framework of Industrie 4.0 has yielded several key findings:

*Process Layer Framework:*We have categorized WAAM processes into distinct layers (workpiece, assembly, and product), each with unique timeframes, stakeholder interactions, and data infrastructure requirements. This layered approach enables more targeted solutions for different aspects of the manufacturing process.

*Security Challenges and Solutions:* We identified layer-specific security and privacy challenges in WAAM environments:

- At the *workpiece layer*, industrial production devices require hardened configurations, secure communication, and proper management of security secrets to protect against external threats.
- The *assembly layer* demands interoperability among stakeholders through secure-by-design protocols, security gateways, and collaborative security configurations.
- The *product layer* requires reliable and trustworthy information exchange, which can be achieved through authentication mechanisms and privacy-preserving computing approaches.

*Data-Driven Quality Control:* In light of the ongoing digital transformation, we demonstrated a practical application of WAAM digitalization through data-driven quality control, which:

- Leverages digital shadows as lightweight models for specific process aspects
- Creates crucial interfaces to production networks and the envisioned World Wide Lab
- Enables closed-loop quality control during the manufacturing process
- Achieved significant reductions in welding fume emissions (12–40%)
- Supports connected applications with FER sensors to minimize exposure and energy consumption

*Implementation Gap:* We identified a critical gap between theoretical Industrie 4.0 concepts and their practical implementation in additive manufacturing, highlighting the need for more comprehensive real-world deployments and evaluations.

*Standards Adoption:* Our findings emphasize the importance of adopting existing standards (IEC 62443, ISO/IEC 27001, NIST SP 800-82) while recognizing that maintaining security is an ongoing process requiring continuous assessment and adaptation, particularly in light of the shift toward interconnected production sites.

These findings contribute to the advancement of WAAM technology by providing both a theoretical framework and practical implementations that enhance process efficiency, product quality, and workplace safety while addressing the security and privacy challenges inherent in connected manufacturing environments.

## Data Availability

All data that has been generated or analyzed as part of this research is either embedded in this published article or part of the supplementary data.
